# Median and ulnar nerve injuries in cyclists: A narrative review

**DOI:** 10.37796/2211-8039.1143

**Published:** 2021-12-01

**Authors:** Dinesh C. Sirisena, Shauna H-S Sim, Ivan Lim, Vaikunthan Rajaratnam

**Affiliations:** a Department of Orthopaedics and Sports Medicine, Khoo Teck Puat Hospital, Singapore; b Yong Loo Lin Medical School, National University of Singapore, Singapore

**Keywords:** Cycling, Median nerve, Ulnar nerve, Compressive neuropathy, Ultrasound, Screening

## Abstract

Cycling is popular internationally as a mode of transport and sport. Cyclists often report sensory and motor changes in the hands during rides. In the past, assessment of these symptoms was based on clinical history, physical examination and neurophysiology. The aim of this narrative review was to evaluate existing publications and determine if there are areas for further improvement in the clinical setting.

**Methods:**

Searches were undertaken in accordance with the PRISMA guidelines using four online databases: PUBMED, OVID, CINAHL and WEB OF SCIENCE. Articles were evaluated using adapted versions of guidelines for case and cohort studies.

**Results:**

2630 articles were found and 13 were included in the review. 2 considered median, 9 considered ulnar and 2 assessed both nerves. 11 were case and 2 were cohort studies. 7 discussed neurophysiology and 1 mentioned ultrasound as a modality of investigation. Interventions were described in 3 articles.

**Conclusion:**

The quality of evidence is generally low when considering this problem. Clinical assessment and neurophysiology are commonly regarded as the method for assessing nerve symptoms amongst cyclists. Advances in musculoskeletal ultrasound add to our early investigative repertoire and may help expedite management and limit future disability. In addition, further research is required into screening and preventative measures amongst cyclists.

## 1. Introduction

Peripheral nerve injuries are common in athletes, occurring anywhere in the body and usually due to direct nerve trauma, sudden traction [1], or a compressive force [2]. Cycling is an increasingly popular activity that people have adopted as transportation, a pastime, or sport. It is estimated that over 7 million people cycle in the United Kingdom weekly [3], while there are nearly 800,000 commuter cyclists daily in the United States [4]. In Singapore, a government survey estimated that 125,000 people cycle regularly [5]. A variety of subcategories (including road, mountain, cyclo-cross, BMX, or track) cater to individual preferences, each with a unique environment [6,7] and associated physical challenges, yet the mechanics and biomechanics remain constant [8]. Primarily intended for directional change and guiding the bicycle, the rider controls the front of the frame via the handlebars. The seat and pedals are rear contact points that carry the rider and produce propulsion [9]. The rider must effectively manage all these intrinsic aspects and also navigate various environmental obstacles.

While cycling is an excellent way of keeping fit and healthy [10–12], it can lead to various injuries from an acute incident or chronic exposure to riding. Common areas where symptoms develop include the back, knees, and shoulders, particularly if the bicycle has not been correctly adjusted to the rider [13]. Cycling may also cause nerve injuries, leading to impotence in male riders [14]. However, the hands are more commonly affected, with injury to the median and ulnar nerves (M&UN), resulting in sensory alterations such as numbness (paresthesia), pain, and muscle atrophy. Dubbed “cyclist’s palsy” [15], this condition can occur acutely, following a single prolonged episode of cycling or after repeated exposure with excessive pressure on the handlebars from a wrong frame size, poor positioning, or overall conditioning of the rider [16]. Often dismissed by cyclists as “part of the ride,” the increasing popularity of this activity will likely mean that cyclist’s palsy will be a more frequent pathology in the future. It is important that rehabilitation specialists, sports physicians, and hand surgeons who encounter riders with these symptoms be aware of the problem and treat it accordingly.

This review aims to summarize existing clinical knowledge of cyclist’s palsy, including investigations that confirm the changes and appropriate treatments to limit the functional impact on the individual.

## 2. Methods

A literature search of the medical databases PubMed, Ovid, CINAHL, and Web of Science was performed following the Preferred Reporting Items for Systematic Reviews and Meta-Analyses (PRISMA) [17]. The following MeSH terms (bicyclist, median nerve, ulnar nerve, median neuropathy, and ulnar neuropathy) were incorporated with additional synonyms (Cyclist, Cycling, and Bicycling) (Table 1) and executed using a Boolean search (Cyclist **OR** Bicyclist **OR** Cycling **OR** Bicycling) **AND** (Median nerve **OR** Ulnar nerve **OR** Median neuropathy **OR** Ulnar neuropathy).

### 2.1. Review process

Articles were imported into Endnote X9.3.3 (Clarivate Analytics, Philadelphia, PA, USA), duplicates were eliminated, and two study members (DS and SS) examined titles and abstracts for suitability. Full texts identified in the screening were then evaluated before disagreements were resolved by consensus. Authors IL and VR further evaluated the screened articles. References of relevant papers were checked for additional studies.

### 2.2. Inclusion and exclusion criteria

Seminal articles that included the search criteria were chosen and formed the foundation for other papers that cited them. Since this was a narrative review, any studies that described M&UN injury, involvement, or neuropathy with cycling were included. All non-cycling-related nerve articles were excluded.

Inclusion criteria were studies with human subjects over 18 years, articles focused on assessing median or ulnar nerve pathologies in cyclists, and articles published in English in peer-reviewed journals. Non-peer-reviewed journals and review articles, anatomical studies, and studies that do not focus on median or ulnar nerve symptoms, did not perform research in cyclists, or had no abstract or English translation were excluded.

### 2.3. Study analysis

Articles were analyzed according to which nerve (median, ulnar, or both) was considered, the type of riding of the subjects, if any inciting event had precipitated the symptoms, the assessments or investigations undertook, interventions that were required, and the key findings from the study. The level of evidence for each article was graded according to the Oxford CEBM Levels of Evidence [18].

### 2.4. Quality assessment

A quality assessment of included articles was undertaken using an adapted form of the National Heart, Lung, and Blood Institute (NHLBI) guidelines for cohort and case studies/series [19].

## 3. Results

**Fig. 1:**
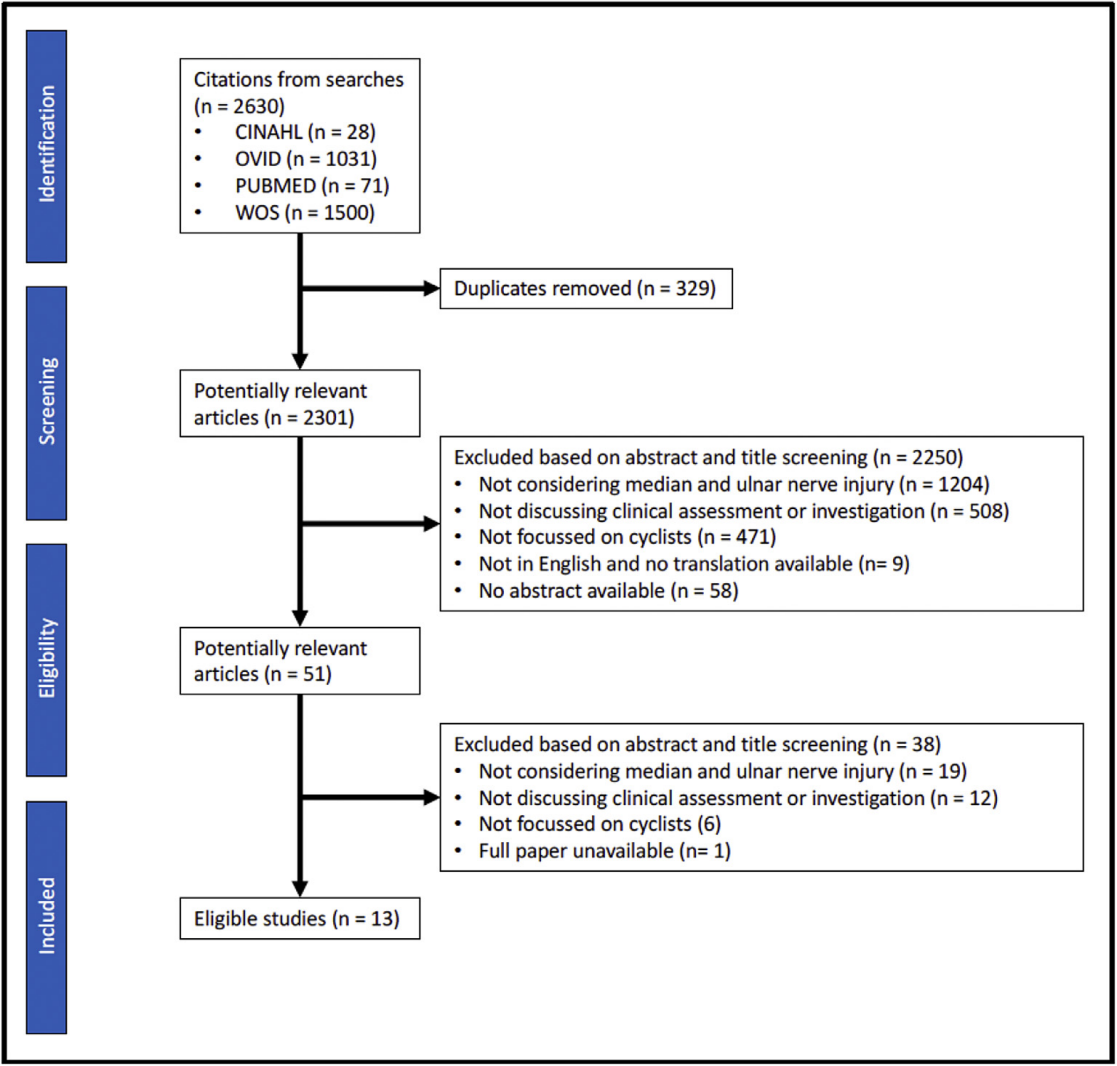
Outline of the review process following PRISMA guidelines 17.

A total of 2630 articles were found in the initial search; 2301 articles were screened based on title and abstract, from which 51 full texts were obtained. A final list of 13 articles was included in the review [16,20–31] (Fig. 1).

### 3.1. Median nerve (Table 2, Section 1)

Isolated median nerve (MN) injuries were infrequently described in the literature. Two case studies reported bilateral symptoms in men following long-distance rides [26,30]. One patient underwent an ultrasound and neurophysiology assessment, while the other did not undergo an initial assessment; however, both received cortisone injections as part of their management.

### 3.2. Ulnar nerve (Table 2, Section 2)

Nine studies described ulnar nerve (UN) injuries. These comprised 4 case reports [20,22,27,31] and 5 case series [21,23–25,28] with 18 cases involved (14 male and 4 female). Bilateral symptoms were described in three [20,23,24], whereas the others described unilateral cases [21–25,27,28,31]. Only two reported hand dominance [21,22]. Eight papers reported the precipitating event before the onset of symptoms; six described single long-distance rides of varying distances [20–23,25,28], of which one included a downhill ride [28]. One article reported multiple long-distance rides [24], and one documented that symptoms developed following a 3- to 4-h bicycle lesson [31]. The mechanism of injury was not described in one study [27]. In six out of nine studies, nerve conduction studies were used as an assessment [20–22,24,28,31], and no other modalities were described. One case series reported hand therapy as a mode of treatment [27]; again, no other intervention was described in the other cases.

### 3.3. Median and UN (Table 2, Section 3)

Two cohort studies evaluated M&UN symptoms in cyclists [16,29]. The first studied 25 riders, randomly selected out of 1800 who participated in a 600-mile cycling event [16]. Undertaking a mixture of strength and sensory assessments, the researchers found that up to 22% of mountain bikers and 25% of those riding road bikes had a reduction in grip strength. However, there were also concurrent UN symptoms with weakness in pincer strength in many of these cases. Only four subjects (16%) reported sensory changes in an MN distribution, but three had concurrent changes in a UN distribution. No further assessments (imaging or neurophysiology studies) were performed. A subsequent investigation was performed among 14 riders who participated in a week-long ride where 70 miles were covered daily [29]. Using an electrophysiological assessment of the hands, they found that three subjects had changes consistent with carpal tunnel syndrome pre-ride, which worsened after their participation. One rider developed these changes de novo following the event.

### 3.4. Quality assessment

Based on the NHLBI [19] guidelines, each of the case studies/series included in the review scored 8/12 (Table 3), while the cohort studies scored 7/11 (Table 4).

## 4. Discussion

This review yielded two case studies focusing on the MN [26,30], nine case studies/series on the UN [20–25,27,28,31], and two cohort studies [16,29] focused on the M&UN. In both articles considering the MN, preexisting pathologies were suspected, and due to protracted symptoms following presentation that were not solved with conservative management, a cortisone injection was required.

The two cohort studies investigating M&UN injuries examined the prevalence in road and mountain bikers [16,29]. The first considered neurophysiological changes in cyclists’ hands and found that conduction was impaired in the first dorsal interossei (FDI) and the abductor digiti minimi (ADM) after the ride. At the same time, several subjects had MN changes.

Although neurophysiology was reported in most articles, ultrasound was used to evaluate the nerves more comprehensively [32–34]. Indeed, only one case report documented the use of ultrasound imaging when assessing the MN [30], leading to the suspicion that chronic changes, in keeping with carpal tunnel syndrome, had been present for some time. Typically, when nerves are examined, they can be assessed at the site of compression, for flattening or pinching of the nerve, and more proximally, where there is swelling suggestive of nerve compression [35]. In the hand, the UN can be traced from Guyon’s canal to its deep and superficial divisions. Measurements and typical pathological changes such as the “notch sign” can help assess and monitor the nerve [36]. As ultrasound imaging resolution improves, it may be possible to assess the nerve glide in a longitudinal plane to identify tethering areas from the compression pathology. Presently, ultrasound is more commonly used to guide treatments around nerves that have been compressed and are causing pain [37]. Thus, ultrasound can improve symptoms and allow subjects to return to normal activities, as in case reports of isolated MN compression [26,30]. However, ultrasound may have a role in screening and tracking the progression of nerve changes in cyclists to identify if and when further assessment is needed. Such progression tracking has been effective in other nerve-related [38] and muscle-related pathologies [39].

The classical description of a “handlebar palsy” [40] involves the UN and its deep branches distal to Guyon’s canal [41]. It is associated with sensory, motor, and coordination aberrations in the hand that can manifest during or several days after the ride. Most studies of this phenomenon were case reports [20,22,27,31] or case series [21,23–25,28]. The subjects mostly presented motor weakness in the FDI or ADM following prolonged compression during riding. Most of the subjects were experienced road-bike riders, with only two articles reporting this phenomenon in mountain bikers [25,28] and one in a novice cyclist [31]. Two articles also described this injury in semi-professional or competitive cyclists [22,28].

Rauch et al. (2016) [42] examined the UN in relation to the hamate in various riding positions using magnetic resonance imaging. They found that with the wrist extended or in ulnar deviation, the nerve was closer to the hamate, representing the common handlebar position adopted by riders. However, owing to the non-dynamic nature of MRI scanning and the cost for the patient, this would not be considered a first-line tool and might only be required with atypical symptoms.

During the ride, pressure on the hands to support the rider’s body weight and subsequent compression of the nerves appears to be the underlying trigger in most cases, particularly during a long ride. Indeed, compression is recognized as a risk factor for acute and chronic neuropathies [43] and has also been reported in occupationally induced nerve injuries such as carpal tunnel syndrome [44,45]. The typical forward riding position, particularly in road cycling, means that handlebar compression can vary according to the gripping style of the cyclist and whether they use gloves. While road cyclists appear to be most commonly affected at the wrist, tri-athletes who place the load on the forearms suffer compression neuropathies at the elbow [46].

Therefore, two interventions are proposed to support the cycling community. The first is incorporating neural protection and preservation among cyclists through education and training to alleviate potential problems in the future. As part of this process, it is important to undertake further research with experts in bicycle biomechanics to investigate different grip types [47], handlebar design, seat height [48], stem length [9], and the use of gloves [47,49], all of which influence weight distribution on the hands. Equally, cycling ergonomics and core muscle training [50] to ensure maintenance of posture should be included more widely in educational resources.

As a further intervention, a screening program for cyclists is proposed, incorporating self-administered questionnaires like the Boston Carpal Tunnel Questionnaire [51], which has been investigated as a potential screening tool for carpal tunnel syndrome in a potentially high-risk cohort [52]. Should symptoms be identified, referral to a specialist for further assessment and study can be recommended.

## 5. Conclusion

Given the rising popularity of cycling internationally, healthcare professionals and cyclists must work together to raise awareness of median and ulnar neuropathies among this active population. Preventive measures, prompt diagnosis, and treatment can potentially limit the morbidity associated with these pathologies. This evaluation highlights the need for further work, particularly when screening cyclists. Hopefully, this work will enable positive symptoms to be identified early rather than attributed to being part of the cycling experience.

## Figures and Tables

**Table 1:** MeSH and additional terms used for the literature search.

MeSH terms	Additional synonyms
Bicyclist	Cyclist
Median nerve	Cycling
Ulnar nerve	Bicycling
Median neuropathy	
Ulnar neuropathy	

**Table 2:** Overall findings from the study with key findings and evidence levels based on the 2011 Oxford CEBM Evidence Levels of Evidence [18].

Author	Study type	Type of cyclist	Inciting event	Number/gender	Nerves considered	Primary assessment	Other assessments	Results	Interventions	Evidence level
**Section 1: Median nerve**										
Braithwaite [26]	Case report	Road	100-mile ride	1 male	Median	Clinical assessment	None	***Unilateral*** Clinical assessment Decreased pin-prick sensation in median nerve distribution of affected hand and weakness of thumb opposition. No obvious muscle wasting.	Cortisone injection is given to relieve symptoms	4
Ali, Delamont, Jenkins, Bland, Mills [30]	Case report	Road	300-km ride	1 male	Median	Clinical assessment	Neurophysiology and ultrasound	***Bilateral*** Clinical assessment Bilateral weakness of thumb abduction and opposition (MRC Grades 2–3), with no weakness of ulnar and radial innervated muscles. No muscle wasting, with sensation preserved. Neurophysiological testing Normal ulnar motor and sensory responses Normal median sensory responses Markedly reduced compound muscle action potential (CMAP) amplitude from abductor pollicis brevis (APB) bilaterally, worse on the right side on motor studies. Distal latency to APB: normal on the right, marginally prolonged on the left. Electromyography (EMG) showed florid fibrillation potentials and positive sharp waves in both APB muscles. There were no voluntary motor units detected. Ultrasound of median nerves at the wrist No focal enlargement of the median nerve suggestive of carpal tunnel syndrome (CTS).	None	4
**Section 2: Ulnar Nerve**										
Eckman, Perlstein, Altrocchi [20]	Case report	Road	3000-mile ride	1 male	Ulnar	Clinical assessment	Nerve conduction studies	***Bilateral*** Right abductor digiti minimi (ADM) Distal latency: 5.9 ms (prolonged) Right 1st dorsal interosseous (DI) Distal latency: 6.9 ms (prolonged) Left ADM Distal latency: 4.4 ms (prolonged) Left 1st DI Distal latency: 4.7 ms (prolonged)	None	4
Noth, Dietz, Mauritz [21]	Case series	Road	400 –2000-km rides	3 male and 1 female	Ulnar	Clinical assessment	Nerve conduction studies	**Case 1** ***Unilateral*** Conduction studies Polyphasic action potential (AP) was recorded over the left hypothenar eminence (surface electrode), which was reduced to 500 μV (right hand 8.5 mV), and the distal latency was prolonged to 10.2 ms (righthand 3.2 ms) **Case 2** ***Unilateral*** On presentation Near-complete paresis of the right-hand muscles innervated by the ulnar nerve. CMAP recorded from the hypothenar eminence Almost completely abolished on the right hand Normal on the left hand Five weeks after presentation Motor function had recovered slightly Increased hypothenar compound AP (3.5 mV in contrast to 8 mV on the left hand).	None	4
Frontera [22]	Case report	Road	20-km ride	1 male	Ulnar	Clinical assessment	Nerve conduction studies	***Unilateral*** Left ADM Distal latency: 3.7 ms Left 1st DI Distal latency: 5.4 ms 4.0 ms (4 weeks after onset)	None	4
Haloua, Collin, Coudeyre [23]	Case series	Road	100–700-km rides	2 male and 1 female	Ulnar	Clinical assessment	None	**Case 1** ***Unilateral*** Clinical assessment Pure right ulnar motor paralysis with interosseous muscle wasting. No sensory impairment. EMG Isolated motor impairment involving all right intrinsic hand muscles innervated by the ulnar nerve. **Case 2** ***Bilateral*** Clinical assessment Left claw hand associated with paresthesia of both hands. Claw hand disappeared in 5 days. EMG Complete blockade of motor and sensory in the left hand and sensory blockade in the right. **Case 3** ***Bilateral*** Clinical assessment Paresthesia of both hands with motor disturbances mainly in the left hand.	None	4
Hankey, Gubbay [24]	Case series	Road	Daily cycling, up to 2–4 h per day	1 male and 1 female	Ulnar	Clinical assessment	Nerve conduction studies	**Case 1** ***Bilateral*** Right 1st DI Distal motor latency: 5.2 ms (prolonged) EMG: Denervation Left ADM Distal motor latency: 4.3 ms (borderline) EMG: Denervation **Case 2** ***Unilateral*** Right ADM Distal motor latency: 4.8 ms (prolonged) EMG: Denervation Right 1st DI Distal motor latency: 7.8 ms (prolonged) EMG: Denervation Right adductor pollicis EMG: Denervation	None	4
Maimaris, Zadeh [25]	Case series	Mountain and road	120–195-km rides	2 male	Ulnar	Clinical assessment	None	**Case 1** ***Unilateral*** Left hand Mild clawing of little finger Marked weakness in ADM Weakness in abduction and adduction of all fingers Positive Froment’s sign Right hand No neurological deficits **Case 2** ***Unilateral*** Left hand Diminished pin-prick sensation in the little finger Decreased power of abduction and adduction on the little finger Froment’s sign mildly positive Right hand No neurological deficits	None	4
Brandsma [27]	Case report	Road	Not stated	1 male	Ulnar	Clinical assessment	None	***Unilateral*** Clinical assessment Paralysis of the intrinsic ulnar muscles with the development of a claw hand bilaterally	Hand therapy and evaluation muscle with strength testing	4
Capitani, Beer [28]	Case series	1 road and 2 mountain	5000-km ride and downhill riding	3 male	Ulnar	Clinical assessment	Nerve conduction studies	**Case 1** *Unilateral* Sensation preserved in the right hand Right fist dorsal interosseus (FDI) Distal latency: 14.1 ms (prolonged, N: ≤4.2 ms) Low amplitude of compound muscle action potential (CMAP) Right abductor digiti minimi (ADM) Conduction studies normal **Case 2** *Unilateral* Sensation preserved in both hands Left FDI Distal latency: 5.4 ms Reduction of CMAP amplitude (1.2 mV) with denervation changes Left ADM Distal latency: 3.0 ms (normal: < 3.3 ms) Normal CMAP amplitude (9.0 mV) **Case 3** *Unilateral* Sensation preserved in both hands. Moderate weakness and atrophy of left ulnar intrinsic hand muscles, except left ADM. Left FDI Distal latency: 5.6 ms (prolonged) Slight reduction of amplitude (3.2 mV) with denervation changes Left ADM Distal latency: 3.0 ms (normal) Normal CMAP amplitude (18.1 mV)	None	4
Selçuk, Kurtaran, Yildirim, Değirmenci, Akyüz [31]	Case report	Leisure	3–4-h bi-cycle lesson	1 female	Ulnar	Clinical assessment	Nerve conduction studies	***Unilateral*** Left FDI Distal latency: 4.7 ms (prolonged) Small amplitude (2.2 mV)	None	4
**Section 3: Median and Ulnar Nerve**										
Patterson, Jaggars, Boyer [16]	Cohort	16 road and 9 mountain	600-km ride	13 male and 12 female	Ulnar and median	Clinical assessment for motor and sensory changes	None	**Motor symptoms** Decrease in grip strength Mountain bikers (M): 22.0%, road bikers (R): 25.0% Decrease in pinch strength M: 38.9%, R: 31.5% Positive resisted abduction test M: 16.7%, R: 25.0% Froment’s sign M: 16.7%, R:12.5% **Sensory symptoms** Tinel’s sign M: 16.7%, R: 3.1% Phalen’s signM: 5.6%, R: 3.1% Elbow provocative test M: 5.6%, R: 3.1% Decreased sensation to distal 5th finger M: 33.0%, R: 3.1%	N/A	3
Akuthota, Plastaras, Lindberg, Tobey, Press, Garvan [29]	Cohort	Road	420-mile tour	7 female 7 male	Ulnar and median	Nerve conduction studies	None	Distal motor latencies of the deep branch of the ulnar nerve to the FDI were prolonged after the event (p < 0.05). Three subjects had CTS before that worsened after the event, and one developed CTS changes in the median nerve after the event.	N/A	3

**Table 3:** Case study quality assessment using the NHLBI guidelines for cohort and case studies/series [19].

Author	Was the study question or objective clearly stated?	Was the study population clearly and fully described, including a case definition?	Were the cases consecutive?	Were the subjects comparable?	Was the intervention clearly described?	Were the outcome measures clearly defined, valid, reliable, and implemented consistently across all study participants?	Was the length of follow-up adequate?	Were the statistical methods well described?	Were the results well described?	Score
Braithwaite [26]	Yes	Yes	No	Yes	No	Yes	No	No	Yes	8
Ali, Delamont, Jenkins, Bland, Mills [30]	Yes	Yes	No	Yes	No	Yes	No	No	Yes	8
Eckman, Perlstein, Altrocchi [20]	Yes	Yes	No	Yes	No	Yes	No	No	Yes	8
Noth, Dietz, Mauritz [21]	Yes	Yes	No	Yes	No	Yes	No	No	Yes	8
Frontera [22]	Yes	Yes	No	Yes	No	Yes	No	No	Yes	8
Haloua, Collin, Coudeyre [23]	Yes	Yes	No	Yes	No	Yes	No	No	Yes	8
Hankey, Gubbay [24]	Yes	Yes	No	Yes	No	Yes	No	No	Yes	8
Maimaris, Zadeh [25]	Yes	Yes	No	Yes	No	Yes	No	No	Yes	8
Brandsma [27]	Yes	Yes	No	Yes	No	Yes	No	No	Yes	8
Capitani, Beer [28]	Yes	Yes	No	Yes	No	Yes	No	No	Yes	8
Selçuk, Kurtaran, Yildirim, Değirmenci, Akyüz [31]	Yes	Yes	No	Yes	No	Yes	No	No	Yes	8

**Table 4:** Cohort study quality assessment using the NHLBI guidelines for cohort studies [19]. (Q1: Was the research question or objective in this paper clearly stated? Q2: Was the study population clearly specified and defined? Q3: Was the participation rate of eligible persons at least 50%? Q4: Were all the subjects selected or recruited from the same or similar populations (including the same time period)? Were inclusion and exclusion criteria for the study prespecified and applied uniformly to all participants? Q5: Was a sample size justification, power description, or variance and effect estimates provided? Q6: For the analyses in this paper, were the exposure(s) of interest measured before the outcome(s) being measured? Q7: Was the time frame sufficient so that one could reasonably expect to see an association between exposure and outcome if it existed? Q8: For exposures that can vary in amount or level, did the study examine different levels of exposure as related to the outcome (e.g., categories of exposure or exposure measured as a continuous variable)? Q9: Were the exposure measures (independent variables) clearly defined, valid, reliable, and implemented consistently across all study participants? Q10: Was the exposure(s) assessed more than once over time? Q11: Were the outcome measures (dependent variables) clearly defined, valid, reliable, and implemented consistently across all study participants? Q12: Were the outcome assessors blinded to the exposure status of participants? Q13: Was loss to follow-up after baseline 20% or less? Q14: Were key potential confounding variables measured and adjusted statistically for their impact on the relationship between exposure(s) and outcome(s)?).

Author	Q1.	Q2.	Q3.	Q4.	Q5.	Q6.	Q7.	Q8.	Q9.	Q10.	Q11.	Q12.	Q13.	Q14.	Score
Patterson, Jaggars, Boyer [16]	Yes	Yes	Yes	Yes	No	No	No	No	Yes	Yes	Yes				7
Akuthota, Plastaras, Lindberg, Tobey, Press, Garvan [29]	Yes	Yes	Yes	Yes	No	No	No	No	Yes	Yes	Yes				7
